# A Review of Catecholamine Associated Cardiomyopathies and Channelopathies

**DOI:** 10.7759/cureus.6957

**Published:** 2020-02-11

**Authors:** Pooja Sethi, Craig D Peiris

**Affiliations:** 1 Cardiology, Texas Tech University Health Sciences Center, Lubbock, USA

**Keywords:** catecholamine, cardiomyopathies, channelopathies

## Abstract

Conditions associated with states of catecholamine toxicity are pheochromocytoma, paraganglioma, Takotsubo’s cardiomyopathy, and catecholaminergic polymorphic ventricular tachycardia. This article defines these conditions along with relevant diagnostic and prognostic factors. The role of catecholamines in these conditions are compared and contrasted. Emphasis is given to the role of endothelial dysfunction as a possible etiologic factor in cardiomyopathy associated with excess catecholamines.

## Introduction and background

Epinephrine, norepinephrine, and dopamine constitute the three catecholamines. The cardiac effects of catecholamines have been well recognized and described extensively in the literature. All three catecholamines have been used for the treatment of hypotension in critically ill patients. However, the excessive exposure of human myocardium to catecholamines has long been recognized as having toxic effects such as inducing myocardial necrosis [1]. Catecholamine-induced cardiomyopathy is a well-recognized and potentially life-threatening complication in pheochromocytoma and paragangliomas [2-3]. Indeed, catecholamines have been postulated to play a critical role in the pathogenesis of Takotsubo cardiomyopathy (TC) [4-5].

Interestingly, lunar catecholamine cardiomyopathy has been postulated as a result of catecholamine elevations in astronauts Neil Armstrong and Irwin [6]. The toxic effects of catecholamine excess while primarily reported in adults can also occur in children. The role of catecholamine-induced cardiac dysfunction has received more attention recently. However, the extent of this phenomenon remains under-recognized. A variety of states associated with a surge of catecholamines have been linked to cardiac dysfunction. These acute increases in catecholamines include accidental or excessive exogenous use of epinephrine and states such as subarachnoid hemorrhage [7]. In this review, we explore cardiomyopathies, channelopathies, and vasculopathies associated with catecholamines excess.

## Review

Cardiomyopathy has been reported as a phenomenon in pheochromocytoma with a prevalence of 11 % [2]. The cardiomyopathy observed in this study was similar to that seen in TC [2]. Although the myocardial dysfunction seen in pheochromocytoma may be a global or focal abnormality.

Catecholamines exert inotropic, dromotropic, and chronotropic effects on the heart. Additionally, catecholamines have vasoconstrictive properties leading to an increase in afterload on the heart. Systemic net vasoconstriction results in increased myocardial oxygen demand. Placing the myocardium at risk for conditions that compromise nutrient delivery. States of catecholamine excess may even compromise blood delivery through effects on the microvasculature of the heart [8]. There are many proposed mechanisms of catecholamines cardiotoxicity.

The microvascular dysfunction caused by catecholamines has been proposed as part of the unifying mechanism of TC [4]. This fact is supported by the evidence that the administration of adenosine, a potent vasodilator, temporarily resolves perfusion and wall motion abnormalities in TC [8]. TC microvascular dysfunction may be mediated through catecholamine-induced endothelial cell destruction making the vessels more prone to spasm [9]. The catecholamine microvascular dysfunction may be a key component of catecholamine cardiomyopathy. Endothelial dysfunction has been observed in TC patients’ years after the initial diagnosis [10]. Microvascular-induced myocardial ischemia may also explain the mild elevation of cardiac enzymes seen in TC.

Other catecholamine-induced outcomes in pheochromocytoma and paraganglioma include a dilated cardiomyopathy and hypertrophic cardiomyopathy [11]. These uncommon cancers also increase the risk of stroke, cardiac arrhythmias, and aortic dissection. Also, adrenergic receptor activation and effects from catecholamine degradation byproducts can all contribute to adverse cardiac effects.

A complete reversal of catecholamine cardiomyopathy has been reported within eight days of pheochromocytoma removal [12]. However, irreversible loss in cardiac function following surgery for pheochromocytoma does occur. In these cases of permanent loss of cardiac function, concurrent coronary artery disease or other comorbidities might be contributory as well.

Exogenous infusion of norepinephrine has been reported to induce myocardial necrosis and leukocyte infiltration with degenerative changes [5]. Many mechanisms have been considered to explain the toxic effects of catecholamines. One hypothesis is that catecholamines increase oxygen-derived free radicals leading to tissue injury and myocyte. Catecholamines may also directly impact the myocardium resulting in enhanced (cAMP-mediated) calcium influx into cardiac muscle promoting necrosis, inflammation, and subsequent fibrosis.

TC is receiving more attention as a novel form of acute heart failure. Typically, apical ballooning of the ventricles resembling a Japanese octopus pot, hence the name, is seen. Most studies report the frequency of TC between 1-4% in patients presenting with the acute coronary syndrome (ACS). Individuals may experience a recurrence of TC. Regional variations in cardiac involvement exist, and biplane left ventriculography may identify focal TC [13]. Right ventricular hypokinesis occurs in approximately 33% of cases and correlates with the severity of left ventricular wall motion abnormalities but does not predict the development of hypotension or shock [14]. As such, mechanisms other than reduced cardiac output are likely involved.

Important risk factors for TC include female sex and neurological and psychiatric disorders [15]. TC is often precipitated by physical or psychological stress and induces a regional change in left ventricular function in the absence of coronary artery obstruction. The presentation of TC is very similar to ACS [16]. Prompt recognition of this condition is important to avoid unnecessary thrombolysis. Patients often have initial elevation in troponins similar to ACS but tend not to demonstrate a progressive increase over time [16]. The marked elevation of catecholamine degradation products indicates that plasma catecholamine levels are elevated significantly above those seen in ACS [5]. Coronary artery disease may be present in patients with TC, but its prevalence and severity are significantly less compared to patients with ACS. 

Several efforts have been made to differentiate this condition from ACS. Highly sensitive Troponin T to creatine kinase MB ratio may provide some synergy to discriminate between TC and ACS [17]. The InterTAK Diagnostic Score determines the probability TC from ACS with high sensitivity and specificity [15]. More research is required to further assess InterTAK's diagnostic ability [15]. The ratio between cardiac enzymes released during events of TC and ACS are likely different [17]. The combination of InterTAK, new research in cardiac enzymes, and clinical judgment hold promise for the identification of TC without cardiac catheterization.

Increased cardiac sympathetic activity due to a variety of stressful events such as acute emotional changes, adrenergic excess states have been implicated as etiologic factors. A wide range of triggers including influenza, anti-influenza vaccination, and hypoglycemia have been implicated as etiologic factors [18]. Exaggerated sympathetic stimulation is probably central to the cause of this syndrome. Intravenous catecholamines can precipitate TC disorder in humans [19]. Sympathetic innervation is greatest at cardiac base and density of beta-adrenoreceptors highest at the apex of the heart. Significant endothelial dysfunction as judged by flow-mediated vasodilation studies can be seen in TC along with increased sympathetic muscle activity [10]. The endothelial dysfunction seen in TC may create hyperreactivity to catecholamines precipitating the condition.

Prognosis is excellent with early diagnosis and treatment. Renin-angiotensin system inhibitors may reduce episodes of TC recurrence [20]. A high prevalence of malignancy has been reported in TC patients and may influence outcomes [21].

Catecholaminergic polymorphic ventricular tachycardia (CPVT) is a rare inherited channelopathy associated with an enhanced risk of arrhythmia and death [22]. If untreated, CPVT is highly lethal, as approximately one-third of affected individuals experience at least one cardiac arrest and many more experience syncopal spells. The initial manifestation may be a cardiac arrest. The mean age of onset is 7-12 years although onset may be as late as 40 years. Initial presenting symptom may be syncope, the condition is difficult to diagnose due to the multiple conditions that lead to syncope with a normal electrocardiogram (EKG). Syncope and EKG changes related to adrenergic stimulation in the absence of a cardiac structural explanation suggests CPVT. CPVT is associated with bradycardia, which may be a useful tool in distinguishing it from long QT syndrome [23].

A major cause of CPVT is autosomal dominant mutations in the cardiac ryanodine receptor channel (RYR2) [23]. Mutations in the RYR2 receptor are not unique to CPVT and can occur in long QT syndrome [23]. Autosomal recessive mutations in the calsequestrin 2 protein gene is another cause of this syndrome. This mutation has variable penetrance and results in defective intracellular calcium metabolism. Increased cytosolic calcium release from the sarcoplasmic reticulum, enhances intracellular calcium levels promoting delayed depolarization and triggered activity. This pattern has some analogy with digoxin, and similar to digoxin toxicity, one of the primary abnormal rhythms is a bidirectional tachycardia [24]. One study found an association between the age of presentation of CPVT and specific gene mutations [25].

Catecholamines can cause these ryanodine-related channels to release calcium in diastole triggering life-threatening arrhythmias - the frequency and the complexity of the ventricular ectopy increase with the workload. Monomorphic premature ventricular complexes may be initially seen followed by bigeminy and subsequently doublets, triplets, bidirectional ventricular tachycardia to polymorphic ventricular tachycardia. These arrhythmias may be provoked during exercise testing and may degenerate into ventricular fibrillation. It should be noted that CPVT can be frequently linked to aberrant sinus node function and may also contribute to atrial tachyarrhythmias [24].

Patients should void vigorous exercise and consider genetic testing for mutations. The use of b-blockers and flecainide are accepted treatments for this disorder. Digoxin should not be used as there is at least one case of it precipitating cardiac death [25]. Implantable cardioverter-defibrillator placement may be needed, and cardiac sympathetic denervation can also be considered [25].

In cases of TC, the use of catecholamines for acute circulatory support may have deleterious effects and their use is associated with increased mortality [4, 26]. Therefore, the need for circulatory support should be balanced with a potential causative role of catecholamines in TC.

## Conclusions

A state of catecholamine excess should be considered in patients presenting with an acute presentation of cardiac decompensation in the absence of ischemic or valvular etiologies. The InterTAK diagnostic score may be a useful tool in managing clinical suspicion for TC in these patients. The diagnosis of pheochromocytoma is complicated by the use of catecholaminergic agents in intensive care settings. This complication necessitates a heavier emphasis on imaging for pheochromocytoma diagnosis. A computed tomography or magnetic resonance imaging of the abdomen may be required to confirm the diagnosis of pheochromocytoma in this setting. Invasive pressure monitoring should be considered in states of catecholamine excess to manage the risk of catecholamine cardiomyopathy.
